# Effects of the Tui-na and Thai Massage Techniques on Vascular Arterial Compliance in Middle-aged Korean Men

**Published:** 2018-02

**Authors:** So-Hyung KANG, Il-Kon KIM, Eun-Ju CHOI, Wi-Young SO

**Affiliations:** 1. Dept. of Sports and Wellbeing, Arts & Sports, Hanyang University, Ansan-si, Korea; 2. Dept. of Sports and Health Management, Mokwon University, Dae-jeon, Korea; 3. Dept. of Physical Education, Daegu Catholic University, Gyeongsan, Korea; 4. Sports and Health Care Major, Korea National University of Transportation, Chungju-si, Korea

## Dear Editor-in-Chief

The Tui-Na and Thai massage techniques have been used widely over a long period in Eastern culture. Tui-Na is a traditional Chinese therapeutic massage technique and has been used since 2700 BC (1). Thai massage is derived from Tui-Na massage in China and Ayurveda massage in India, and has been practiced unchanged for 1000 yr (2).

The term “massage” indicates pressing, rubbing, and scrubbing using the hands, and massage therapy is used for injuries, musculoskeletal pain, and correction of body shape. The Tui-Na and Thai massage techniques have been used not only to treat musculoskeletal conditions but also to improve circulation of the blood and metabolism, increase physical fitness, and to treat several diseases (3, 4). However, no scientific study has thoroughly evaluated the effects of massage on vascular compliance. The present study investigated the effects of the Tui-Na and Thai massage techniques on vascular compliance using a non-invasive method, i.e., pulse wave velocity (PWV) (5).

Thirty healthy middle-aged men (aged approximately 40 yr) attended the Sports Medicine Laboratory at Mokwon University in Daejeon-si, Korea, for assessment of vascular compliance. They were divided into a Thai massage group (n=10), a Tui-Na massage group (n=10), and a control group (n=10) (Table 1).

**Table 1:** Characteristics of the study subjects

***Characteristic***	***Tui-Na massage group (n=10)***	***Thai massage group (n=10)***	***Control group (n=10)***	***F***	***P***
Age (yr)	45.10±1.85	45.50±2.17	45.30±2.79	0.075	0.928
Height (cm)	168.70±2.91	169.30±4.11	172.30±3.56	2.934	0.070
Weight (kg)	73.60±2.88	73.70±2.45	73.60±3.37	0.004	0.996
Body fat (%)	25.40±1.96	25.10±2.18	25.60±1.27	0.186	0.831
Heart rate (beat/min)	71.10±3.48	70.60±2.76	66.90±14.44	0.692	0.509
Systolic blood pressure (mmHg)	120.10±3.64	122.10±3.35	122.40±3.57	1.263	0.299
Diastolic blood pressure (mmHg)	78.30±4.72	80.30±5.01	83.50±5.80	2.549	0.097

Data are presented as means±standard deviations

One-way analysis of variance was used.

Massages were administered three times a week for 40 min each over a period of 8 wk by four massage experts, each with more than 10 yr of experience. For Tui-Na massage, the subjects were positioned straight on the massage table. Hand manipulations, including rolling (Gun Fa), the one-finger pushing method (Yi Zhi Chan Fa), and scrubbing (Ca Fa), were then performed at a frequency of 120 times per minute from the head to the feet, including the neck, shoulders, arms, chest, abdomen, and legs. For Thai massage, the subjects were positioned straight on the massage table. The massage expert then pressed along the Sen path (the energy in China and Prana in India) using the feet, palms, thumbs, and knees, with equal pressure applied to the left and right sides of the body. The massage was performed in the supine and prone positions. The massage concluded in the sitting position, and was followed by stretching. The control subjects were instructed to perform normal daily activities and to avoid certain physical activities.

Vascular compliance was assessed by PWV using an approved medical device (PWV-3.0, KM-Tech, Seoul, Korea). Electrocardiography leads applied to the left and right forearms were linked to the device with the subject in the supine position, and PWV measurements were performed when heart rate and blood pressure were confirmed to be stable. PWV was divided by subject height to control for individual differences in vascular length and calculated before and after massage therapy.

All data are presented as the mean±standard deviation. One-way analysis of variance was performed to compare the physical characteristics of the subjects and changes in vascular compliance in the left and right forearms between the study groups. Tukey's post hoc test was also performed. All analyses were performed using SPSS ver.18.0 (IBM Corp., Armonk, NY). Statistical significance was set at *P*<0.05.

The changes in vascular compliance in the left and right forearms are presented in Table 2.

**Table 2:** Changes in vascular compliances of the left and right forearms

***Group***	***Right forearm vascular compliance (pre)***	***Right forearm vascular compliance (post)***	***Change (post–pre)***	***F***	***P***	***Left forearm vascular compliance (pre)***	***Left forearm vascular compliance (post)***	***Change (post–pre)***	***F***	***P***
Tui-Na massage(n=10)	268.69±6.02	281.61±5.97	12.93±8.63^###^	20.00	<0.001 ^***^	272.94±4.91	282.69±5.12	9.75±3.87^###^	32.89	<0.001 ^***^
Thai massage(n=10)	267.93±8.74	282.97±9.47	15.04±5.88^###^			270.00±8.70	283.51±6.97	13.51±5.27^###^		
Control(n=10)	272.25±5.59	269.26±6.90	−2.99±6.04			273.22±6.85	271.10±8.45	−2.12±4.24		

Data are presented as means±standard deviations

One-way analysis of variance with Tukey’s post-hoc test was used.

### *P*<0.001 vs. control;

*** *P*<0.001

Vascular compliance was significantly better on both sides in the Tui-Na and Thai massage groups than in the control group (*P*<0.001). However, there were no significant differences in vascular compliance between the Tui-Na and Thai massage groups (*P*>0.05).

Massage using either the Tui-Na or the Thai technique has a positive effect on vascular compliance. These massage techniques have similar efficacy.
